# Clinical analysis of 547 patients with neuroendocrine tumors in a Chinese population: A single‐center study

**DOI:** 10.1002/cam4.2259

**Published:** 2019-05-24

**Authors:** Lijie Song, Xuejia Zhai, Shunli Yu, Yihui Ma, Feng Wang, Xuxu Yu, Shuang Tao, Yujin Lian, Minjie Yang, Weili Tao, Qingxia Fan

**Affiliations:** ^1^ Department of Oncology The First Affiliated Hospital of Zhengzhou University Zhengzhou China; ^2^ Department of Urology The First Affiliated Hospital of Zhengzhou University Zhengzhou China; ^3^ Department of Pathology The First Affiliated Hospital of Zhengzhou University Zhengzhou China

**Keywords:** epidemiology, neuroendocrine tumors, pathology, prognosis, treatments

## Abstract

**Background:**

Neuroendocrine tumors (NETs) are rare, which has resulted in a lack of published data on their epidemiology and clinical features. We therefore aimed to investigate the epidemiology, clinical features, treatments, and prognosis of patients with NETs.

**Methods:**

The clinicopathologic characteristics of 547 patients who were pathologically diagnosed with NETs were retrospectively analyzed, including age, sex, primary and metastatic sites, symptoms, pathology, treatment, and prognosis.

**Results:**

The 547 patients had a wide age range (9‐87 years), with a male to female ratio of 1:1.1. The primary tumor sites included 413 in the digestive system, 74 in the lung, 15 in the mediastinum, 8 in unknown sites, and 37 in other sites. Of the 413 patients with digestive system NETs, the pancreas, rectum, and stomach were the most common primary sites. Blood metastases were found in 84 patients at initial diagnosis, and the liver, bone, and lung were the most frequent sites of metastasis. Lymph node metastases were found in 82 patients at initial diagnosis. Surgery and chemotherapy were the most widely applied treatments. Statistical analysis showed that age <50 years, female sex, lower‐grade tumor, no distant metastasis, intestinal NET and surgery indicated a favorable prognosis.

**Conclusions:**

A difference between China and other countries is that small intestinal NETs are quite common in other countries but are rare in China. In China, the most common primary sites are the pancreas, rectum, and stomach. Furthermore, no unified treatments exist, though prognoses could be improved by using methods such as surgery, targeted therapies, and somatostatin analogs.

**Clinical Trial Registration:**

This study was not a clinical trial.

## 1 INTRODUCTION

Neuroendocrine neoplasms (NENs) are a heterogeneous group of tumors originating from peptidergic neurons and neuroendocrine cells; these tumors can be divided into neuroendocrine tumors (NETs), neuroendocrine carcinomas (NECs), and mixed neuroendocrine/nonneuroendocrine neoplasms (MiNENs).^1^, ^2^ NETs are rare with a low incidence, which has contributed to a deficiency in large epidemiologic studies of patients with this disease. However, the Surveillance, Epidemiology, and End Results (SEER) program showed an increase from 1.09 new cases per 100 000 in 1973 to 6.98 per 100 000 in 2012, an increase in 540%.^3^ The United States, Norway, Japan, and South Korea have established similar databases. However, only single‐center reports have been published, the number of cases is small, and survival analyses in China are scarce.

## 2 MATERIALS AND METHODS

### 2.1 Research objective

In all, 547 patients with a pathological diagnosis of NET who presented at the First Affiliated Hospital of Zhengzhou University between January 2011 and April 2018 were enrolled. Patients with medullary thyroid carcinoma, pheochromocytoma, and paraganglioma were excluded. Relevant case data were obtained from the pathology database of the First Affiliated Hospital of Zhengzhou University. This study was approved by the Ethics Committee of the First Affiliated Hospital of Zhengzhou University. The pathologic diagnosis of all cases in this study was assessed by two professional pathologists.

### 2.2 Pathological diagnosis

The pathological diagnosis of gastroenteropancreatic neuroendocrine tumors (GEP‐NETs) was based on the World Health Organization (WHO) Classification of Tumors of Endocrine Organs, which was published in 2017,^2^ the Consensus on Pathological Diagnosis of Gastroenteropancreatic Neuroendocrine Tumors in China^1^ was referenced to classify NETs into G1, G2, and NET‐G3 grades. The pathological diagnostic criteria for lung and mediastinal NETs were based on the WHO Classification of Tumors of the Lung, Pleura, Thymus, and Heart,^4^ which divides these NETs into typical carcinoid and atypical carcinoid tumors. The clinical staging of NETs was performed according to the American Joint Committee on Cancer Cancer Staging Manual, 8th edition.^5^

### 2.3 Statistical analysis

SPSS statistical software version 22.0 (SPSS Inc, Chicago, IL, USA) was used to analyze data. Normally distributed continuous variables are expressed as the mean and standard deviation, and significant differences between groups were assessed with independent samples *t* tests. Categorical variables were analyzed using the Chi‐square test. The Kaplan‐Meier method was employed for survival analysis, and log‐rank tests were applied for comparisons among groups. When calculating the survival rate, the specified end point event was NET‐related death. Survival at the end of the follow‐up period was recorded as censored data. Statistical significance was assessed by two‐tailed tests with an α level of 0.05.

## 3 RESULTS

### 3.1 Clinical information

Among the 547 patients with a pathological diagnosis of NET at the First Affiliated Hospital of Zhengzhou University between January 2011 and April 2018, the age range was 9‐87 years, the average age was 50.2 ± 13.8 years, the peak incidence age group at diagnosis was 50‐59 years, and the sex ratio (male to female) was 1:1.1 (265/282). The proportions of age ranges were as follows: 161 patients were 50‐59 years old, accounting for 29.3%; 143 patients were 40‐49 years old, accounting for 24.9%; and 100 patients were 60‐69 years old, accounting for 19.3%. The average ages of males and females were 51.1 ± 14.1 and 49.3 ± 13.5 years, respectively, and the difference was not statistically significant (t = 1.5, *P* = 0.1).The average diameter of the primary tumor was 2.7 ± 3.0 cm (range, 0.1‐20.4 cm), as based on postoperative resection specimens or imaging examinations performed before surgery.

### 3.2 Primary tumor sites

Of all NETs, 413 were located in the digestive system, 74 were pulmonary, 15 were mediastinal, 8 were of unknown primary origin, and 37 were located in other sites (Table 1). Of the 413 digestive system NETs, the pancreas, rectum, and stomach were the most common sites, and the patients with NETs in the duodenum, liver, appendix, gallbladder and common bile duct, jejunum/ileum, colon, and esophagus comprised a relatively small proportion of all patients (Table 1). Sixty‐one patients had insulinoma, and two patients had VIPoma.

**Table 1:** Clinicopathologic characteristics of the study population

	N (%)
Primary site	
Total	547
Gastroenteropancreatic	392 (71.7)
G1	205 (52.3)
G2	145 (37.0)
NET‐G3	42 (10.7)
Pancreas	141 (25.8)
G1	53 (37.6)
G2	78 (55.3)
NET‐G3	11 (7.8)
Rectum	136 (24.9)
G1	99 (72.8)
G2	29 (21.3)
NET‐G3	7 (5.1)
Stomach	81 (14.8)
G1	40 (49.4)
G2	24 (29.6)
NET‐G3	17 (21.0)
Duodenum	19 (3.5)
G1	8 (42.1)
G2	7 (36.8)
NET‐G3	4 (21.1)
Appendix	7 (1.3)
G1	4 (57.1)
G2	3 (42.9)
NET‐G3	0
Colon	4 (0.7)
G1	0
G2	2 (50.0)
NET‐G3	2 (50.0)
Jejunum/ileum	4 (0.7)
G1	1 (25.0)
G2	2 (50.0)
NET‐G3	1 (25.0)
Liver	15 (2.7)
Gallbladder and common bile duct	5 (0.9)
Esophagus	1 (0.2)
Pulmonary	74 (13.5)
Typical	39 (52.7)
Atypical	35 (47.3)
Mediastinum	15 (2.7)
Unknown	8 (1.5)
Other sites	37 (6.8)
Ki‐67 index	
≤2%	247 (45.2)
2%‐20%	258 (47.2)
20%‐60%	42 (7.7)

Abbreviation: NET, neuroendocrine tumor.

### 3.3 Common metastatic sites

Blood metastases were found in 84 (15.4%, 84/547) patients at initial diagnosis. The most frequent site of distant metastasis was the liver (75.0%, 63/84), followed by bone (21.4%, 18/84) and the lungs (16.7%, 14/84). Among the 84 NETs with blood metastases, 45 were GEP‐NETs with liver metastases, three were lung NETs with liver metastases, two were lung NETs with lung metastases, two were adrenal NETs with lung metastases, six were lung NETs with bone metastases, four were rectal NETs with bone metastases, and four were mediastinal NETs with bone metastases. Lymph node metastases were found in 82 (15.0%, 82/547) patients at initial diagnosis. Nineteen were lung NETs and 50 were GEP‐NETs, and of these, 8 were rectal NETs, 20 were gastric NETs, and 17 were pancreatic NETs.

### 3.4 Initial symptoms

Patients with different primary tumor sites have different initial symptoms. Common symptoms in patients with lung NETs were cough and hemoptysis (31.1%, 23/74) and blood in the sputum (28.4%, 21/74). Common symptoms of rectal NETs were abdominal pain, abdominal distension (27.2%, 37/136), blood in the stool (13.2%, 18/136), and changes in stool habits (12.5%, 17/136). Most patients with stomach NETs had abdominal pain and abdominal distension (50.6%, 41/81). Among the pancreatic NETs, 61 were insulinomas, which were characterized by hypoglycemia (100%, 61/61). Patients with nonfunctional pancreatic NETs had abdominal pain and abdominal distension (36.3%, 29/80).

### 3.5 Pathologic features

Among the 392 patients with GEP‐NETs, which accounted for 94.9% of all patients with digestive system NETs, the most common tumor grade was G1, followed by G2 and NET‐G3 (Table 1). Among the 74 patients with pulmonary NETs, 39 had typical carcinoid tumors, and 35 had atypical carcinoid tumors (Table 1). Of the 547 cases of NETs, 355 (64.9%, 355/547) were stage I/II, 50 (9.1%, 50/547) were stage III, and 84 (15.4%, 84/547) were stage IV. Fifty‐eight cases (10.6%, 58/547) could not be staged. Of the 547 NETs stained for Ki‐67, a Ki‐67 index ≤2% accounted for 45.2%, an index >2% and ≤20% accounted for 47.2%, and an index >20% and ≤60% accounted for 7.7% (Table 1). The positive rates of immunohistochemical staining for synaptophysin, CD56 and chromogranin A were 97.8% (523/535), 84.7% (444/524), and 41.9% (173/413), respectively.

### 3.6 Treatment methods

Of the 547 patients, approximately two‐thirds (64.4%, 352/547) underwent surgery, of whom 20.7% (73/352) underwent endoscopic surgery. Fifty‐three (7.5%) of the 547 patients were treated with chemotherapy and other medical treatments, of whom 34 (6.2%, 34/547) received an etoposide plus platinum regimen, 8 (1.5%, 8/547) received a temozolomide and capecitabine regimen, 4 (0.7%, 4/547) received a temozolomide and tegafur regimen, 2 (0.4%, 2/547) received a temozolomide regimen, and 5 (0.9%, 5/547) received octreotide treatment. Thirty‐eight patients (7.0%, 38/547) received postoperative adjuvant chemotherapy, and 95 patients (17.4%, 95/547) received only supportive treatment (some patients received multiple treatments); targeted therapy and radiotherapy were rarely used. Of the 63 patients with NETs that metastasized to the liver, six patients underwent hepatic chemoembolization, eight underwent liver radiofrequency ablation, and five underwent hepatectomy; the remaining underwent resection of the primary lesions or chemotherapy.

### 3.7 Follow‐up and survival

**Figure 1:**
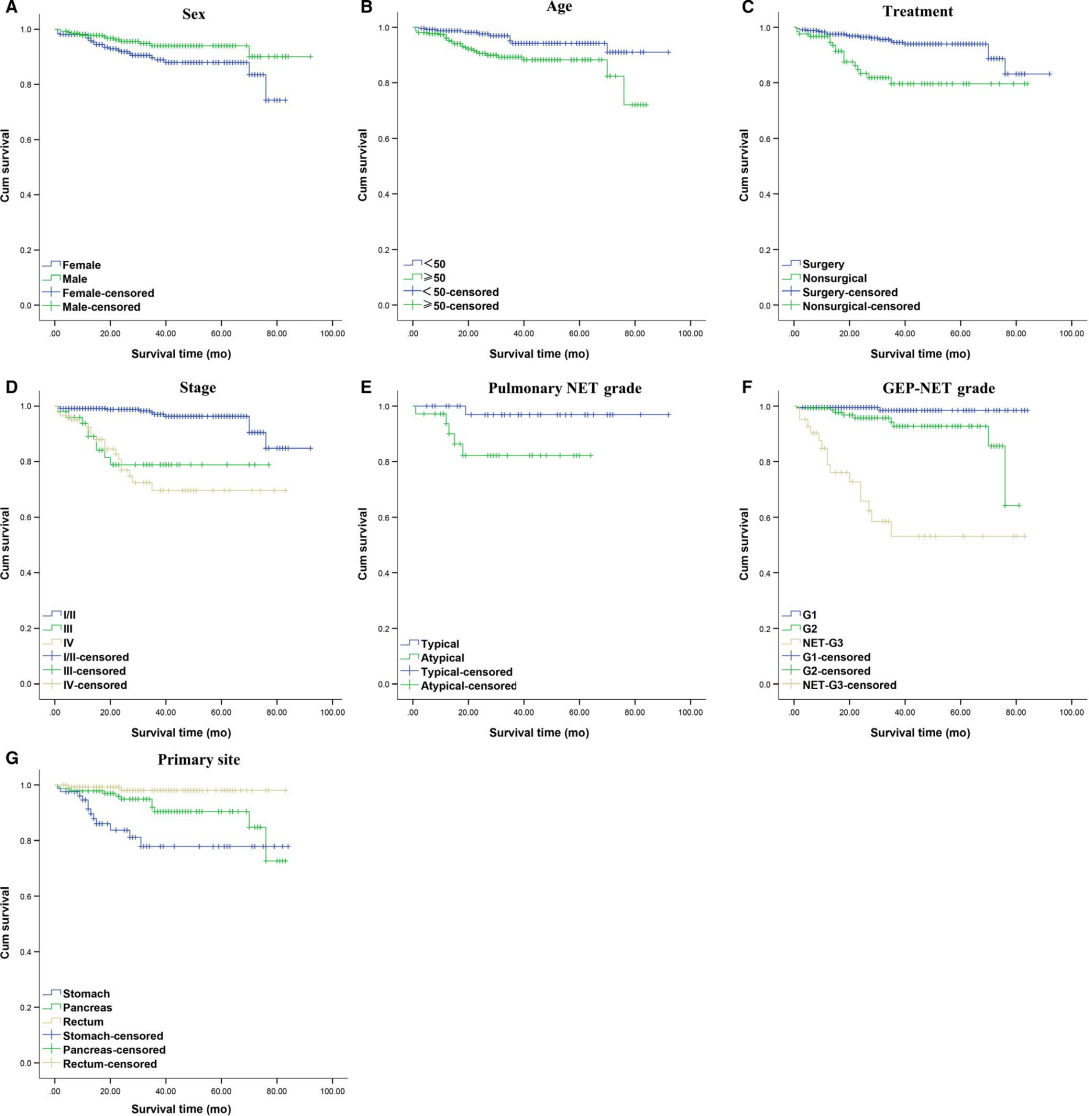
Kaplan‐Meier analysis of overall survival. A, Overall survival stratified by sex of NET patients. B, Overall survival stratified by age of NET patients. C, Overall survival stratified by treatment approach used in NET patients. D, Overall survival stratified by stage of NET patients. E, Overall survival stratified by grade in pulmonary NET patients. F, Overall survival stratified by grade in GEP‐NET patients. G, Overall survival stratified by primary site in NET patients. GEP‐NETs, gastroenteropancreatic neuroendocrine tumors; NET, neuroendocrine tumor

All patients were followed up by telephone. The duration was from the date of pathological diagnosis to the date of death or the last follow‐up, which was on September 5, 2018. The follow‐up rate was 72.4%, and 151 patients were lost to follow‐up. The overall median survival time of the patients with NETs was not observed during the observation period due to the relatively short follow‐up period and low mortality of patients with NETs. The relationships between age, sex, stage, grade, treatment or tumor size and prognosis of patients with NETs were analyzed. The statistical analysis showed that an age <50 years, female sex, lower grade, lack of distant metastasis, intestinal NETs, and surgery were associated with a favorable prognosis; in contrast, tumor size was not related to prognosis (data are shown in Table 2, and survival curves are shown in Figure 1).

**Table 2:** Survival analysis of the study population stratified by clinicopathological characteristics

Clinicopathological characteristic	Total (N)	Dead (N)	Surviving (N)	χ^2^	*P*
Sex				4.446	0.035
Male	265	25	240		
Female	282	13	269		
Age				7.469	0.006
<50	247	10	237		
≥50	300	28	272		
Stage				45.4	<0.01
I/II	355	11	344		
III	50	9	41		
IV	84	18	66		
Treatment				14.839	<0.01
Surgery	425	21	404		
Nonsurgical	122	17	105		
GEP‐NETs				75.025	<0.01
G1	205	2	203		
G2	145	9	136		
NET‐G3	42	15	27		
Pulmonary				4.228	0.04
Typical	39	1	38		
Atypical	35	5	30		
Primary site				16.503	<0.01
Stomach	81	12	69		
Pancreas	141	11	130		
Rectum	136	2	134		
Size of primary tumor				3.626	0.163
≤2cm	283	21	262		
2‐4cm	46	0	46		
≥4 cm	122	8	114		
Tumor size in the stomach				3.742	0.154
≤2 cm	43	8	35		
2‐4 cm	13	0	13		
≥4 cm	15	1	14		
Tumor size in the pancreas				2.528	0.283
≤2 cm	82	8	74		
2‐4 cm	24	0	24		
≥4 cm	13	1	12		
Tumor size in the rectum				3.421	0.181
≤2 cm	73	0	73		
2‐4 cm	20	1	19		
≥4 cm	24	1	23		
Tumor size in the lung				1.02	0.796
≤2 cm	34	3	31		
2‐4 cm	7	0	7		
≥4 cm	16	1	15		

Abbreviations: GEP‐NETs, gastroenteropancreatic neuroendocrine tumors; NET, neuroendocrine tumor.

## 4 DISCUSSION

NETs have been consistently considered rare tumors,^6^ but a significant increase in the incidence of NETs in the United States,^3^ Norway,^7^ and China^8^, ^9^, ^10^ has been reported in recent publications. A large sample size of autopsy reports from Sweden showed that the annual incidence of NETs between 1958 and 1969 was 8.4 per 100 000, which is much higher than the incidence rate in clinical reports from the same period; this indicates that many NETs may remain asymptomatic and have no clinical significance.^11^ Our group studied 547 patients with NETs. The male to female ratio was 1:1.1, which is not in agreement with the rate reported by Chi et al^8^ (1.9:1) and Taiwan^12^ (1.6:1), as both studies reported more men than women with this tumor. However, the ratio is consistent with that in a report by Qiu et al^9^ (1.1:1) and data from Norway^7^ (1.1:1) and the United States^3^ (1.1:1), in which the numbers of male and female patients are essentially the same. The average patient age was 50.2 ± 13.8 years, and the peak incidence age group at diagnosis was 50‐59 years. These findings corresponded to the results of Qiu et al,^9^ who reported that peak disease incidence occurred at 51‐60 years of age, while Chi et al^8^ reported that peak disease incidence occurred in an older age range of 60‐69 years; similarly, a peak disease incidence at 65 years or older was reported in the United States.^3^ The United States,^3^ Taiwan,^12^ and Chi et al^8^ reported case data before 2012, but Qiu et al^9^ and this study used records collected after 2011. Therefore, the difference in the peak incidence age group may be related to the improvement in people's health awareness, the continuous improvement of endoscopic technology, imaging and other examination methods, and the application of immunohistochemical staining, which allows asymptomatic patients to be detected early. It was found that the average diameter of the primary tumor was 2.7 ± 3.0 cm and that tumor size was not related to prognosis. However, Chi et al^13^ and Pasaoglu et al^14^ reported that a larger tumor diameter results in a worse prognosis, and this finding may be related to the research objective. The research objective of the present study is to distinguish NETs that do not include NEC. Since the prognosis of NETs is good and the sample size of the present study is insufficient, the relationship between tumor size and prognosis cannot be elucidated. Our follow‐up analysis showed that the 1‐, 3‐, and 5‐year survival rates of females were higher than those of males, and this difference was statistically significant (*P* = 0.04). The 1‐, 3‐, and 5‐year survival rates of patients younger than 50 years were higher than those age 50 years or older, and the difference was statistically significant (*P* < 0.01).

The low incidence of NETs has contributed to the deficiency in large epidemiologic studies of patients with this disease. Therefore, this study retrospectively analyzed the clinical data of 547 patients with pathologically confirmed NETs treated at the First Affiliated Hospital of Zhengzhou University between January 2011 and April 2018 and explored patient epidemiological characteristics, clinical features, treatment methods, and prognosis. Dasari et al^3^ analyzed 64 971 cases of NENs (excluding small cell lung cancer) from 1973 to 2012 in the US SEER database. The top four primary sites in these patients were the lung (1.49/100 000), small intestine (1.05/100 000), rectum (1.04/100 000), and pancreas (0.48/100 000). Boyar et al^7^ studied a total of 16 075 patients with NENs from 1993 to 2010 in the Cancer Registry of Norway (CRN) database. The top five primary sites in these patients were the lung (6.1%), small intestine (5.9%), appendix (4.2%), pancreas (2.8%), and colon (1.9%). Tsai et al^12^ studied 2187 patients with NENs (excluding small cell lung cancer) in the Taiwan Cancer Registry database from 1996 to 2008, including NENs in the rectum (25.4%), lung (20.0%), stomach (7.4%), pancreas (6.0%), colon (5.3%), and small intestine (5.3%). Ito et al^15^ analyzed GEP‐NEN cases between 2005 and 2010 in Japan, including 2845 pancreatic NENs and 4406 gastrointestinal NENs. The top four primary sites of gastrointestinal NENs were the rectum (55.7%), small intestine (18.9%), stomach (15.1%), and colon (2.1%). Cho et al^16^ analyzed 4951 patients with GEP‐NENs in a multicenter study in Korea from 2000 to 2009. The top five primary sites in these patients were the rectum (48.0%), stomach (14.6%), pancreas (8.7%), colon (7.9%), and small intestine (7.7%). Lim et al^17^ analyzed 125 patients with GEP‐NENs in a single‐center study in South Korea from 2009 to 2011. The top five primary sites in these patients were the rectum (79.8%), duodenum (5.6%), pancreas (4.8%), stomach (3.2%), and colon (2.4%). Cho et al^16^ reported that the incidence of rectal NENs in Korea increased significantly; however, that of NENs in the stomach showed a significant downward trend, and others were stable during 2000‐2009. Lim et al^17^ indicated that the conclusion is certain and has a certain predictive value in Korea. Fan et al^10^ analyzed 2010 patients with GEP‐NENs, including NENs in the pancreas (31.5%), rectum (29.6%), stomach (27.2%), small intestine (5.6%), and colon (3.0%),in a multicenter study in China from 2001 to 2010. Chi et al^8^ analyzed 252 patients with NENs (excluding small cell lung cancer) at the Cancer Hospital of the Chinese Academy of Medical Sciences. The top four primary sites in these patients were the lung (29.4%), rectum (23.0%), stomach (10.7%), and appendix (3.2%). Qiu et al^9^ analyzed 903 patients with NENs (excluding small cell lung cancer) from 2012 to 2016 at China‐Japan Friendship Hospital. The top four primary sites in these patients were the stomach (22.9%), rectum (22.3%), pancreas (20.5%), and lung, thymus or mediastinum (9.6%). Of the 547 patients with NETs in this study, 392 (71.7%) patients presented with GEP‐NETs, and 74 patients (13.95%) presented with lung NETs. The top four primary sites in the GEP‐NET patients were the pancreas (25.8%), rectum (24.9%), stomach (14.8%), and duodenum (3.5%). Similar to other national and international data, in this study, the gastrointestinal system and pancreas were the most common primary sites. The top three most common primary sites in China were the pancreas, rectum and stomach, while in Japan and South Korea, they were the rectum, small intestine and stomach. In the United States, the most common primary sites were the small intestine, rectum, and pancreas, and in Norway, they were the small intestine, appendix, and pancreas. This variation may be related to differences in race, geography, and diet. Differences were also observed in common sites in different centers in China, which may be related to different treatment approaches. The prognosis of NET patients is related to the primary tumor site. The follow‐up analysis showed that the 1‐, 3‐, and 5‐year survival rates of the patients with intestinal NETs were higher than those of the patients with pancreatic NETs and that the survival rates of patients with pancreatic NETs were higher than those of patients with gastric NETs, and the difference was statistically significant (*P* < 0.01).The proportion of NET‐G1 tumors among all rectal NETs was significantly higher than that among the pancreatic and gastric NETs, while the proportion of NET‐G3 tumors among all gastric NETs was significantly higher than that among the rectal and pancreatic NETs. The prognosis of NET‐G1 tumors was significantly better than that of NET‐G2 and NET‐G3 tumors. It can be concluded that the prognosis of rectal NETs is superior to that of pancreatic NETs and gastric NETs, which may be related to the tumor grading proportions (Table 2).

NETs can be functioning or nonfunctioning. Nonfunctioning tumors can be asymptomatic or may present with nausea, abdominal pain, weight loss, intestinal obstruction, and bleeding. Functioning tumors, in contrast, secrete peptides and neurotransmitters, such as serotonin, histamine, and tachykinins, which cause the typical carcinoid syndrome. Functioning tumors can also secrete other hormones, such as insulin, glucagon, parathyroid hormone related peptide, vasoactive intestinal peptide, and growth hormone. Excessive secretion of these hormones can lead to clinical manifestations such as hypoglycemia, hyperglycemia, hypercalcemia, diarrhea, and acromegaly.^18^ The first symptom is closely related to the primary site of disease. The most common symptoms of gastric and rectal NETs are abdominal pain and abdominal distension, and those of lung NETs are cough and hemoptysis. These observations are consistent with other reports from China.^8^

Ki‐67 index labeling and grading have become essential for the prognostic assessment of GEP‐NENs. The latest WHO classification of tumors^2^ changed the Ki‐67 index distinguishing G1 and G2 NETs to 3% and introduced a new category of well‐differentiated neoplasms, NET‐G3, in addition to the previous categories of NET‐G1 and NET‐G2. The differential diagnosis between NET‐G3 (well‐differentiated) and NEC (poorly differentiated) might be difficult, and the authors of the WHO classification therefore suggested the use of a number of immunohistochemical markers to facilitate distinction of the two entities. Even if these changes actually concerned only pancreatic NETs, they would probably be applied to all digestive NETs. NET‐G3 is consistent with the concept of highly proliferative NETs (well‐differentiated, Ki‐67 index more than 20% and <60%) proposed in China in 2013.^1^ Morphological features are still the gold standard for distinguishing subgroups of lung NETs, and the value of the Ki‐67 index in lung NET classification is still being investigated.^19^ Of the 392 patients (71.7%) with GEP‐NETs in this study, 52.3% were G1, 37.0% were G2, and 10.7% were NET‐G3. The follow‐up analysis showed that the 1‐, 3‐, and 5‐year survival rates of G1 patients were higher than those of G2 patients, and that those of G2 patients were higher than those of NET‐G3 patients; this difference was statistically significant (*P* < 0.01).

No uniform solution currently exists for the treatment of NETs. Surgical resection is the preferred treatment for NETs, and generally, for locoregional or recurrent localized NETs, surgical resection should be considered if the majority (more than 90%) of the gross disease can be resected safely.^20^ Primary tumor resection in patients with metastatic NETs is currently controversial, but for patients with clinical symptoms mainly caused by the primary tumor, removal of the primary tumor is recommended.^21^ In this study, 77.7% of the patients underwent surgery. The follow‐up analysis showed that the 1‐, 3‐, and 5‐year survival rates of the patients treated with surgery were higher than those of nonsurgical patients, and the difference was statistically significant (*P* < 0.01).

In patients with inoperable NETs, the goal is to control endocrine‐related symptoms and tumor growth and prolong survival. Nonsurgical treatment options mainly include somatostatin analogs (SSAs), multikinase inhibitors, targeted therapies, chemotherapy and radiolabeled SSAs.^22^ SSAs, including octreotide long‐acting repeatable, lanreotide, and pasireotide, are treatments for NETs at the earliest stage, and as antisecretory and antiproliferative agents, they are mainly used to alleviate the symptoms of carcinoid syndrome and to control tumor growth in symptomatic and asymptomatic, unresectable, high‐tumor burden patients with well‐differentiated NETs. In addition, there is a consensus that SSAs should be used regardless of somatostatin receptor imaging results in patients with carcinoid syndrome.^23^, ^24^, ^25^ Among patients with refractory carcinoid syndrome not adequately controlled by SSAs, telotristat ethyl is the appropriate drug of choice.^23^, ^26^ ENETS consensus guidelines recommend systemic chemotherapy as a different treatment option for pancreatic NETs, as this can be used in G1 and G2 tumors. However, systemic chemotherapy is not recommended for nonpancreatic NETs unless the NETs have a Ki‐67 index >15%, display invasive biological behavior or are negative for somatostatin receptors. In pancreatic NETs, streptozotocin combined with 5‐fluorouracil is an established cytotoxic therapy combination. Since streptozotocin has not been marketed in China, temozolomide in combination with capecitabine is generally used. Temozolomide is a therapeutic option for bronchial carcinoids based on data from a small study.^27^, ^28^ Recently, Crespo et al^28^ reported 65 patients with metastatic or unresectable NETs treated with capecitabine and temozolomide in daily practice in patients in whom the primary tumor location included the pancreas, lungs, stomach, ileum, and jejunum, among others, and showed that the median progression‐free survival of this combination for pancreatic NETs and nonpancreatic NETs was 18.4 and 15.3 months, respectively. We thus have reason to believe that a capecitabine and temozolomide chemotherapy regimen may be a promising and feasible option in terms of efficacy and tolerability for patients with either pancreatic NETs or nonpancreatic NETs. In this study, eight patients received a temozolomide and capecitabine regimen, four received a temozolomide and tegafur regimen, two received a temozolomide regimen, and five received octreotide treatment. Targeted drugs, such as everolimus or sunitinib, may be used as first‐ or second‐line options in patients with pancreatic NETs. Everolimus may also be recommended for nonpancreatic advanced NETs.^29^

Metastasis is the most important factor that affects the prognosis of NET patients. In NET‐G1 and NET‐G2 patients, surgery with curative intent should always be considered, even if liver and/or lymph node metastases are present. Cytoreductive surgery, orthotopic liver transplantation, local ablation, and intraarterial therapy all improve the prognosis of patients with locally advanced or metastatic NETs. More than 50% of patients with GEP‐NENs have been reported to have liver metastases at diagnosis.^30^, ^31^ The optimal treatment sequence for different treatments remains controversial, and most patients have metastatic disease at the time of diagnosis. Therefore, the development of a treatment plan for each patient requires multidisciplinary discussion, and integration of locoregional and systemic approaches to maximally improve patient prognosis is the current treatment trend. In this study, the most common site of NET metastasis was the lymph node, and the most common site of NET blood metastasis was the liver. Of the patients with liver metastases, 71.4% had GEP‐NETs. Of the 63 patients with NETs that metastasized to the liver, six underwent hepatic chemoembolization, eight underwent liver radiofrequency ablation, five underwent hepatectomy; the remainder underwent primary lesion resection or chemotherapy. Other common metastatic sites were the lungs and bones. The follow‐up analysis showed that the 1‐, 3‐, and 5‐year survival rates of the patients without metastasis were higher than those of patients with metastasis, and this difference was statistically significant (*P* < 0.01).

## 5 CONCLUSIONS

In summary, the incidence of NETs is low, the gastrointestinal system and the pancreas are the most common primary sites of NETs, and the prognosis is good. The difference between China and other countries is that small intestinal NETs are quite common in other countries but are rare in China. No uniform solution exists for the treatment of NETs, but surgery is the cornerstone treatment. Nonsurgical treatments include SSAs, targeted therapies, chemotherapy, and radiolabeled SSAs. In addition, China still needs to establish a national NET database to improve our understanding of NETs.

## CONFLICT OF INTEREST

The authors made no disclosures.

## AUTHOR CONTRIBUTIONS

Lijie Song was involved in conceptualization, formal analysis, investigation, methodology, project administration, resources, supervision, writing the original draft, and manuscript review and editing. Xuejia Zhai was involved in investigation, methodology, supervision, writing the original draft, and manuscript review and editing. Shunli Yu was involved in formal analysis and manuscript review and editing. Yihui Ma was involved in conceptualization, methodology, and manuscript review and editing. Feng Wang and Qingxia Fan were involved in conceptualization, investigation, methodology, and manuscript review and editing. Xuxu Yu, Shuang Tao, Yujin Lian, Minjie Yang, and Weili Tao were involved in data curation and manuscript review and editing.

## DATA AVAILABILITY STATEMENT

This article data sharing, hereby declare.

## References

[1] Chinese Pathologic Consensus Group for Gastrointestinal and Pancreatic Neuroendocrine Neoplasm . Chinese pathologic consensus for standard diagnosis of gastrointestinal and pancreatic neuroendocrine neoplasm. Chin J Pathol. 2013;42:691‐694. 21616002

[2] Lloyd R , Osamura R , Klöppel G , et al. Classification of Tumours of Endocrine Organs. 4th ed Lyon: IARC Press; 2017.

[3] Dasari A , Shen C , Halperin D , et al. Trends in the incidence, prevalence, and survival outcomes in patients with neuroendocrine tumors in the United States. JAMA Oncol. 2017;3:1335‐1342. 28448665 10.1001/jamaoncol.2017.0589 PMC5824320

[4] Travis WD , Brambilla E , Burke AP , et al. WHO Classification of Tumors of the Lung, Pleura, Thymus and Heart. 4th ed Vol. 29. Lyon: IARC Press; 2015. 10.1097/JTO.0000000000000663 26291007

[5] Amin MB , Edge S , Greene F , et al. Staging Manual. 8th ed New York: Springer; 2017.

[6] Yao JC , Hassan M , Phan A , et al. One hundred years after "carcinoid": epidemiology of and prognostic factors for neuroendocrine tumors in 35,825 cases in the United States. J Clin Oncol. 2008;26:3063‐3072. 18565894 10.1200/JCO.2007.15.4377

[7] Boyar Cetinkaya R , Aagnes B , Thiis‐Evensen E , Tretli S , Bergestuen DS , Hansen S . Trends in incidence of neuroendocrine neoplasms in Norway: a report of 16,075 cases from 1993 through 2010. Neuroendocrinology. 2017;104:3729‐10. 10.1159/000442207 26562558

[8] Chi Y , Jiang WC , Du F , et al. Neuroendocrine tumors: analysis of 252 cases. Chin J Oncol. 2013;35:67‐70. 10.3760/cma.j.issn.0253-3766.2013.01.015 23648305

[9] Qiu X , Liu M , Liu Q , et al. Analysis of primary site and pathology on 903 patients with neuroendocrine neoplasms. Chin J Gastrointest Surg. 2017;20:993‐996. 28900988

[10] Fan J‐H , Zhang Y‐Q , Shi S‐S , et al. A nation‐wide retrospective epidemiological study of gastroenteropancreatic neuroendocrine neoplasms in china. Oncotarget. 2017;8:71699‐71708. 29069739 10.18632/oncotarget.17599 PMC5641082

[11] Fraenkel M , Kim MK , Faggiano A , Valk GD . Epidemiology of gastroenteropancreatic neuroendocrine tumours. Best Pract Res Clin Gastroenterol. 2012;26:691‐703. 23582913 10.1016/j.bpg.2013.01.006

[12] Tsai H‐J , Wu C‐C , Tsai C‐R , Lin S‐F , Chen L‐T , Chang JS . The epidemiology of neuroendocrine tumors in Taiwan: a nation‐wide cancer registry‐based study. PLoS ONE. 2013;8:e62487. 23614051 10.1371/journal.pone.0062487 PMC3632554

[13] Chi Y , Du F , Zhao H , Wang J‐W , Cai J‐Q . Characteristics and long‐term prognosis of patients with rectal neuroendocrine tumors. World J Gastroenterol. 2014;20:16252‐16257. 25473180 10.3748/wjg.v20.i43.16252 PMC4239514

[14] Pasaoglu E , Dursun N , Ozyalvacli G , Hacihasanoglu E , Behzatoglu K , Calay O . Comparison of World Health Organization 2000/2004 and World Health Organization 2010 classifications for gastrointestinal and pancreatic neuroendocrine tumors. Ann Diagn Pathol. 2015;19:81‐87. 25702616 10.1016/j.anndiagpath.2015.01.001

[15] Ito T , Igarashi H , Nakamura K , et al. Epidemiological trends of pancreatic and gastrointestinal neuroendocrine tumors in Japan: a nationwide survey analysis. J Gastroenterol. 2015;50:58‐64. 24499825 10.1007/s00535-014-0934-2

[16] Cho M‐Y , Kim JM , Sohn JH , et al. Current trends of the incidence and pathological diagnosis of gastroenteropancreatic neuroendocrine tumors (GEP‐NETs) in Korea 2000–2009: multicenter study. Cancer Res Treat. 2012;44:157‐165. 23091441 10.4143/crt.2012.44.3.157 PMC3467418

[17] Lim CH , Lee IS , Jun BY , et al. Incidence and clinical characteristics of gastroenteropancreatic neuroendocrine tumor in Korea: a single‐center experience. Korean J Intern Med. 2017;32:452‐458. 28490723 10.3904/kjim.2015.232 PMC5432789

[18] Xu J , Liang H , Qin S , et al. Expert consensus on Chinese gastrointestinal and pancreatic neuroendocrine neoplasms (2016 edition). Chin Clin Oncol. 2016;21:927‐946.

[19] Garg R , Bal A , Das A , Singh N , Singh H . Proliferation marker (Ki67) in sub‐categorization of neuroendocrine tumours of the lung. Turk Patoloji Derg. 2019;35:15‐21. 30070306 10.5146/tjpath.2018.01436

[20] Kunz PL , Reidy‐Lagunes D , Anthony LB , et al. Consensus guidelines for the management and treatment of neuroendocrine tumors. Pancreas. 2013;42:557‐577. 23591432 10.1097/MPA.0b013e31828e34a4 PMC4304762

[21] Howe JR , Cardona K , Fraker DL , et al. The surgical management of small bowel neuroendocrine tumors: consensus guidelines of the North American Neuroendocrine Tumor Society. Pancreas. 2017;46:715‐731. 28609357 10.1097/MPA.0000000000000846 PMC5502737

[22] Pasricha G , Padhi P , Daboul N , Monga DK . Management of well‐differentiated gastroenteropancreatic neuroendocrine tumors (GEPNETs): a review. Clin Ther. 2017;39:2146‐2157. 29173655 10.1016/j.clinthera.2017.10.010

[23] Strosberg JR , Halfdanarson TR , Bellizzi AM , et al. The North American Neuroendocrine Tumor Society consensus guidelines for surveillance and medical management of midgut neuroendocrine tumors. Pancreas. 2017;46:707‐714. 28609356 10.1097/MPA.0000000000000850 PMC5642985

[24] Caplin ME , Pavel M , Ćwikła JB , et al. Lanreotide in metastatic enteropancreatic neuroendocrine tumors. N Engl J Med. 2014;371:224‐233. 25014687 10.1056/NEJMoa1316158

[25] Wolin E , Jarzab B , Eriksson B , et al. Phase III study of pasireotide long‐acting release in patients with metastatic neuroendocrine tumors and carcinoid symptoms refractory to available somatostatin analogues. Drug Des Devel Ther. 2015;9:5075‐5086. 10.2147/DDDT.S84177 PMC4562767 26366058

[26] Kulke MH , Hörsch D , Caplin ME , et al. Telotristat ethyl, a tryptophan hydroxylase inhibitor for the treatment of carcinoid syndrome. J Clin Oncol. 2017;35:14‐23. 27918724 10.1200/JCO.2016.69.2780

[27] Fine RL , Gulati AP , Krantz BA , et al. Capecitabine and temozolomide (CAPTEM) for metastatic, well‐differentiated neuroendocrine cancers: the Pancreas Center at Columbia University experience. Cancer Chemother Pharmacol. 2013;71:663‐670. 23370660 10.1007/s00280-012-2055-z

[28] Crespo G , Jiménez‐Fonseca P , Custodio A , et al. Capecitabine and temozolomide in grade 1/2 neuroendocrine tumors: a Spanish multicenter experience. Future Oncol. 2017;13:615‐624. 27802780 10.2217/fon-2016-0434

[29] Pavel M , O''Toole D , Costa F , et al. ENETS consensus guidelines update for the management of distant metastatic disease of intestinal, pancreatic, bronchial neuroendocrine neoplasms (NEN) and NEN of unknown primary site. Neuroendocrinology. 2016;103:172‐185. 26731013 10.1159/000443167

[30] Grandhi MS , Lafaro KJ , Pawlik TM . Role of locoregional and systemic approaches for the treatment of patients with metastatic neuroendocrine tumors. J Gastrointest Surg. 2015;19:2273‐2282. 26341823 10.1007/s11605-015-2931-z

[31] Kennedy AS . Hepatic‐directed therapies in patients with neuroendocrine tumors. Hematol Oncol Clin North Am. 2016;30:193‐207. 26614377 10.1016/j.hoc.2015.09.010

